# 

**Published:** 1939

**Authors:** 


### CONTENTS.

| | Page |
|---|---|
| **The Twenty-Eighth Long Fox Memorial Lecture: Forest Folk: Modern Medicine in the Congo.** By C. C. Chesterman, O.B.E., M.D., M.R.C.P. | 223 |
| **Acute Appendicitis.** By H. Chitty, M.S., F.R.C.S. | 241 |
| **War Surgery in Spain.** Review by A. Rendle Short, M.D., B.S., B.Sc., F.R.C.S. | 249 |
| **Reviews of Books** | 252 |
| **Editorial Notes** | 258 |
| **Obituaries:** G. T. Myles, M.R.C.S., L.R.C.P. | 261 |
| A. Fells, M.B., C.M., F.R.C.S.E. | 261 |
| M. F. Staniland, M.R.C.S., L.R.C.P. | 262 |
| **The Medical Library of the University of Bristol** | 263 |
| **Publications Received** | 265 |
| **Local Medical Notes** | 266 |

